# Urinary tract infection in diabetic patients

**DOI:** 10.4103/0971-4065.73429

**Published:** 2010-10

**Authors:** Ramen Kr. Baishya, A. Mathew, D. R. Dhawan, M. R. Desai

**Affiliations:** Department of Urology, Muljibhai Patel Urological Hospital, Nadiad - 387 001, Gujarat, India

Sir,

We read the article “Prevalence of lower urinary tract infection in South Indian type 2 diabetic subjects” by Janifer *et al*. with great interest. Diabetic patients with urinary tract infection is a common scenario in the nephrourology outpatient department. Treating them is a real challenge.[1] Here, we would like to highlight few important points. Firstly, fungal urinary tract infection in diabetic patients might be asymptomatic and should be sought in every diabetic patient with urinary tract infection.[2] Secondly, we feel that ultrasound-guided suprapubic aspiration of urine is useful in those patients with repeated contamination in urine culture report.[3] Thirdly, we believe that a minimum of 2 weeks of antibiotic therapy is necessary as eradication of the organism in diabetic patients is difficult. Improper duration of antibiotic will encourage resistance.[4] Fourthly, circumcision might be beneficial, especially in those patients who cannot maintain personal hygiene. We would like to know the author’s views regarding these points.
